# A Cross-Sectional Study to Assess the Frequency and Risk Factors Associated with Cesarean Section in Southern Punjab, Pakistan

**DOI:** 10.3390/ijerph18168812

**Published:** 2021-08-21

**Authors:** Muhammad Fawad Rasool, Saira Akhtar, Iltaf Hussain, Abdul Majeed, Imran Imran, Hamid Saeed, Muqarrab Akbar, Muhammad Omer Chaudhry, Anees ur Rehman, Waseem Ashraf, Faleh Alqahtani, Hussain Alqhtani

**Affiliations:** 1 Department of Pharmacy Practice, Faculty of Pharmacy, Bahauddin Zakariya University, Multan 60800, Pakistan; saira338akhtar@gmail.com (S.A.); altaf9216@gmail.com (I.H.); abdulmajeed@bzu.edu.pk (A.M.); aneesurrehman@bzu.edu.pk (A.u.R.); 2 Department of Pharmacology, Faculty of Pharmacy, Bahauddin Zakariya University, Multan 60800, Pakistan; Imran.ch@bzu.edu.pk (I.I.); chishtiwaseem@yahoo.com (W.A.); 3 University College of Pharmacy, Allama Iqbal Campus, University of the Punjab, Lahore 54000, Pakistan; hamid.pharmacy@pu.edu.pk; 4 Department of Political Science, Bahauddin Zakariya University, Multan 60800, Pakistan; muqarrabakbar@bzu.edu.pk; 5 School of Economics, Bahauddin Zakariya University, Multan 60800, Pakistan; omer@bzu.edu.pk; 6 Department of Pharmacology and Toxicology, College of Pharmacy, King Saud University, Riyadh 11451, Saudi Arabia; 7 Department of Clinical Pharmacy, College of Pharmacy, Najran University, Najran 61441, Saudi Arabia; hmhalqhtani@nu.edu.sa

**Keywords:** cesarean section, maternal care, risk factors, knowledge, mothers

## Abstract

The increasing frequency of cesarean section (CS) is a major public health issue, and it is on the rise in Pakistan. A cross-sectional study approach was used to assess the frequency of CS and its contributing factors, along with the assessment of knowledge in mothers who had undergone CS in one of the under-developed regions of Pakistan. Data collection was done by using a self-developed study questionnaire. The statistical package for social sciences (SPSS) was utilized for the statistical analysis. During the study period, a total of 173 (69.7%) women have given births by CS; among those, 104 (60.1%) were elective/planned CSs while 69 (39.8%) were emergency CSs. The higher CS frequency was significantly associated with younger age (*p* = 0.03) and pre-term gestational age (*p* < 0.001). Pregnancy complications, such as gestational diabetes, hypertension, preeclampsia/eclampsia, and vaginal bleeding, were the significant risk factors for CS (*p* < 0.001). The highlighted contributing factors to CS in the current study were preterm of gestational age, mothers of a younger age (20–24 years), and mothers that belong to urban populations. These risk factors can be addressed by implementing community-focused educational interventions during the gestational period. The sample size in this study was small; therefore, the results cannot be generalized to the whole population.

## 1. Introduction

Labor pain is often associated with fear and anxiety in women [1,2]. The individual’s perception and attitude toward the labor pain, the definition of labor pain, coping mechanisms against pain, and other associated behaviors greatly affect the decision regarding mode of delivery [3,4]. Several contributing factors have been identified that are related to the increasing trend of cesarean section (CS). These factors include the perception that it is painless and safer, its convenience for mothers and families, and hospital policies promoting CS and discouraging vaginal births after CS [5,6]. In addition, the higher cost of CS is the major factor that is contributing to the increasing rate of CS [7]. The expenses of CS in lower-middle-income countries including Pakistan are very high (2.8–5.0 times) compared to normal vaginal delivery (NVD), but in contrast, this expense is much lower in higher-income countries (1.1–1.8 times) [8,9,10,11]. This high cost of CS encourages hospitals, especially privately owned hospitals, to promote and perform CS [12].

The CS is the most widely used obstetric operation that has significantly contributed to the improvement of obstetric care [13]. Nowadays, CSs are not performed according to the recommendations, but they are perceived as an escape from labor pain by the general population [13,14]. It has been seen that the risk of maternal deaths associated with CS delivery has increased three times as compared to NVD. The most highlighted complications for newborns that are delivered by CS are fetal respiratory problems such as low appearance, pulse, grimace, activity, and respiration (APGAR) score, fetal injury, and childhood asthma [15,16,17].

The increasing trend of CS has been seen worldwide, but it varies from country to country and within countries. The reported rate of CS in developed countries ranges from 23.8% to 50% while in developing countries, this rate is falling to less than 10% [18,19,20]. Moreover, according to the World Health Organization (WHO), CS use is continuing to rise globally, accounting for 21% of all childbirths. It has been predicted that this number could increase to 29% by 2030 [21]. Globally, the reported rate of CS is significantly higher than the rate recommended by the WHO, which advises that the CS rate should be within the limit of 15% or less to balance the associated benefits and risks [22].

Pakistan is included in the top 10 countries, where CSs are accountable for 59% of the global maternal death burden [23]. The basic health indicators reveal that Pakistan is coping with many demographic and health issues, particularly in maternal and child care. Overall, the maternal mortality ratio (MMR) in Pakistan has been reduced from 521 cases per 100,000 population in 1990 to 178 cases per 100,000 population in 2015. Pakistan has not achieved the target of 130 cases per 100,000 population by the year 2015 [22]. Moreover, obstetric and neonatal care services in Pakistan are unacceptable [24]. There are considerable socioeconomic discrepancies in the maternal healthcare system of Pakistan [25]. The rising rate of CS in Pakistan is alarming and a major public health issue. There is a concern about the rising rate of CS in the urban areas of Pakistan, which is approximately 25% [26,27]. Keeping this in view, the present study was conducted to assess the frequency of CS and its contributing factors in the less developed area of Southern Punjab, Pakistan. Additionally, the study also assessed the knowledge of mothers regarding CS at the obstetrics and gynecology department of the hospital.

## 2. Materials and Methods

### 2.1. Study Design and Participants

An observational cross-sectional study was carried out between September 2019 and March 2020. The study was performed at Fatimah Jinnah women’s hospital, which is a 45 bedded facility in Multan, Pakistan. The study population consisted of women that had given birth at the obstetrics and gynecology department of the hospital.

### 2.2. Study Tool

A self-developed questionnaire was designed for data collection. The questionnaire comprised three domains. The first domain was related to sociodemographic factors, including age, gestational age (weeks), education, place of residency, societal life, physical activity, hookah (a local traditional tobacco pipe) user, parity (births given after the 20th week of gestation), and gravidity (the number of confirmed pregnancies, without considering the outcome). The second domain comprised questions regarding obstetric variables, such as pregnancy complications and indications for CS. The last part of the study questionnaire was related to knowledge regarding CS. The study tool can be seen in Supplementary Table S1.

Furthermore, the Royal College of Obstetricians and Gynecologists standard categorization (1–4) was used to assess the urgency for CS [28].

### 2.3. Ethical Considerations and Data Collection

The current study was reviewed and approved by the Ethical Committee of the Department of Pharmacy Practice, Faculty of Pharmacy, Bahauddin Zakariya University, Multan, Pakistan (Reference No: Acad/PRAC/18-20/09). The study was performed as per the declaration of Helsinki. The data were collected by face-to-face interviews. Informed consent was taken from each individual before participation in the study. Fictitious numbers were assigned to each study questionnaire. The study was reported following The Strengthening the Reporting of Observational Studies in Epidemiology (STROBE) guidelines [29].

### 2.4. Statistical Analysis

The statistical package for social sciences (SPSS v25) was used for the statistical analysis. The categorical variables were presented as frequencies and percentages. The Chi-square or Fisher exact test was used for assessing the association between the sociodemographic, obstetric variables, and indications for CS with the cesarean delivery.

## 3. Results

**Figure 1:**
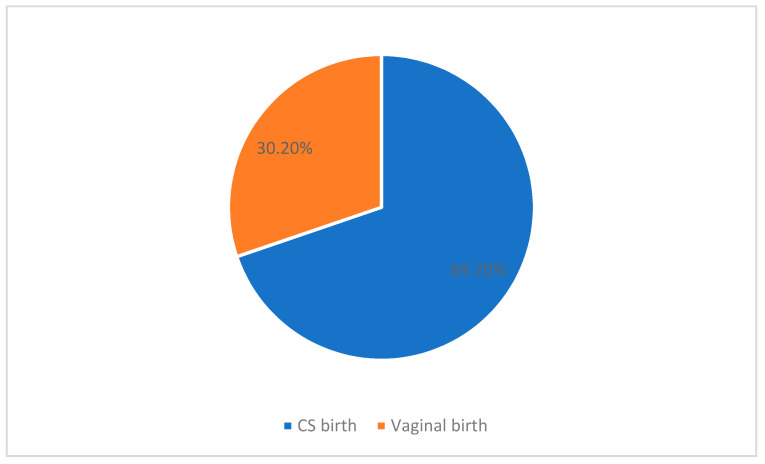
Rate of cesarean section in the study population. *p* < 0.001.

**Figure 2:**
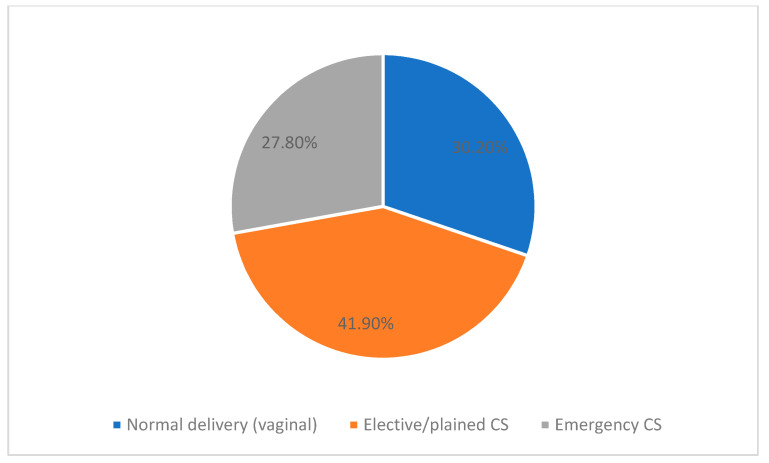
Frequency of delivery mode in the study participants.

In this study, the frequency of CS was 173 out of 248 (69.7%), as shown in Figure 1. Regarding the mode of delivery, the majority of the participants had undergone scheduled/elective cesarean birth (60.1%) while 75 (30%) participants had normal vaginal birth, as shown in Figure 2.

The higher CS frequency was significantly associated with the younger age category (20–24 years) and pre-term gestational age (*p* = 0.03). Moreover, there was a high number of cesarean deliveries in females with low multi-parity (*p* < 0.001). The details of sociodemographic association with the pattern of delivery are given in Table 1.

Among 173 cesarean births, most of the participants had pregnancy complications. The highlighted complications were thyroid problem (complication out of total: 34 out of 36) vaginal bleeding (complication out of total: 13 out of 13), and pre-eclampsia/eclampsia (complication out of total: 13 out of 13). Pregnancy complications including gestational diabetes, hypertension, preeclampsia/eclampsia, and vaginal bleeding were the significant risk factors for CS (*p* < 0.001). The pregnancy complications can be seen in Table 2.

According to the RCOG classification of CS, 19.8% of the participants were from category 1, who needed immediate CS, and 41.1% of the participants were from category 4. The detail can be seen in Table 3.

The majority of the participants were aware of CS (99.5%) and 69.7% had experienced it. Most of the participants did not know the feasibility of vaginal delivery after CS (56.9%). Half the participants were unaware of blood requirements during or after the operation (50%). The participants considered CS dangerous, but the opinion regarding deaths associated with CS was negative (55.6%). All the women said that they would have CS if it is the physician’s recommendation. All the respondents reported that vaginal birth is associated with severe labor pain. The participants’ knowledge regarding CS can be seen in Table 4.

## 4. Discussion

The current study was performed to assess the frequency of CS and its contributing factors along with its knowledge among mothers in Southern Punjab, Pakistan. The current study revealed a higher frequency of CS. The most important contributing factors towards CS were preterm gestational age, younger age (20–24 years), and mothers that belong to urban areas.

The cesarean section rate (CSR) is increasing globally with every passing year, especially in developing countries such as Pakistan. A multiple indicator cluster survey (MICS) shows that the CSR was 3% in 1990–1991, and it increased to 19.10% in 2012–2013 [30]. Results from the current study are consistent with this increasing rate of CSR and the higher frequency of CS (69.7%). Moreover, the reported CSR from other provinces of Pakistan, such as Sindh (17.4%), Khyber Pakhtunkhwa (Kpk) (5.3%), and Balochistan (1.70%), was lower than in our study [31,32]. It is known that, in developed countries, CSR has been increasing, ranging from 14.9% (Israel) to 54.9% (Turkey) as evident from the report of the Organization for Economic Corporation and Development countries (OECD) [33]. This increasing trend of CS is alarming. Therefore, the government and the health regulatory agencies in Pakistan should design and implement community-based interventions to adhere to the WHO recommended rate.

This study demonstrated an increased rate of cesarean birth in urban areas (74.5%) as compared to rural ones (62%). This difference may be attributed to decreased physical activities and limited access to the health infrastructure of urban mothers and rural mothers, respectively, during their gestational periods. Pakistan is a developing country with a less developed health infrastructure, in which the populations of rural areas have limited access to the hospitals; therefore, they have to switch to a normal mode of delivery [34]. However, it is worth mentioning that this may be associated with worse diagnosis and predisposing to CS in rural areas. Similar differences have been reported from Vietnam, where CSR was higher in urban (42.4%) than in rural populations (22.9%) [35]. Increased physical activities during the gestational period reduce the risk of cesarean birth, as good physical activity strengthens the pelvic floor muscles in women which aids in normal vaginal birth [36]. Pakistan has been trying to cope with the problem of maternal care by establishing specialized health units, operating emergency obstetrical care services in health units and implementing a national maternal care program. The implemented national family planning and chief care programs are offering home-based healthcare services for the underserved and rural communities [37]. However, the higher CSR and MMR indicate the ineffectiveness of these programs [22]. Therefore, it is necessary to reshape these programs and the implemented policies to bring the current CSR in line with the recommendations of WHO.

Gestational age may be a contributing factor to cesarean birth, as very preterm singletons and multiples are at high risk of CS, while this risk is lower in singletons at 39 and 40 weeks of gestation [38,39]. Our study showed similar results, where preterm (≤37 weeks) and late-term (41 weeks) pregnancies were linked to an increased risk of cesarean birth. Comparatively, the CSR in the United States (US) showed decreased cesarean births at 38 weeks, but it was greater for birth at 39 weeks [40]. The low gestational age may not directly contribute to the CS, as a slowdown in the progress of labor, the risk of intrauterine asphyxia, and other factors are also predisposed to CS [11]. Specific sub-groups of gestational age could be used by clinicians and health planners to design targeted CS reduction policies.

Pregnancy complications are also responsible for the increasing rate of cesarean delivery [41]. The current study highlighted a few common pregnancy complications, such as gestational diabetes mellitus, hypertension, pre-eclampsia, thyroid disorder, and significant vaginal bleeding. Additionally, other reported promoting factors of CS are fetal grief, absence of labor development, prior cesarean, hypertension, miscarriage, and stillbirth [42,43,44]. These complications can be addressed by promoting maternal care by providing easy access to healthcare facilities, regular follow-ups, and appropriate adherence to the prescribed treatment during pregnancy.

CS, on the one hand, has a positive effect on women’s health and newborn morbidity and mortality, but it also increases the risk of uterine rupture, abnormal placentation, ectopic pregnancy, stillbirth, and premature delivery [45,46]. There is mounting evidence that newborns delivered by CS are subjected to a variety of hormonal, physical, microbiological, and medical exposures, and that these exposures can significantly affect newborn physiology. Short-term consequences of CS include impaired immunological development, a higher risk of allergy, atopy, and asthma, and a reduction in the diversity of the intestinal microbiota [47,48]. Therefore, giving a realistic clinical picture of CS might influence a woman’s decision about her mode of delivery.

All the participants of the current study were willing to perform CS on doctors’ advice. The patient–doctor relationship is the key indicator of patient satisfaction and medical care quality [49,50]. This relationship leads to the belief of patients in doctors which may attribute to the acceptance of doctor advice regarding CS. Clinicians can influence women’s decisions regarding childbirth, as they have the authoritative position and patients trust them [51]. Moreover, the patient’s poor knowledge regarding maternal care and CS may be accounted for by the willingness to perform CS based on doctors’ advice. Additionally, this poor knowledge leads the patient to accept and perform CS without indication. In contradiction with our findings, Nigerian women showed a negative attitude to perform CS on doctors’ advice [52,53]. Moreover, the majority of the participants thought that once they had given birth by CS then, in the future, they could never have an NVD. This is the major myth that has been reported previously [54,55,56]. It has been seen that almost 30–35% of CS performed were due to prior cesarean birth [57]. Patient education is the only key to address such myths and attitudes, and therefore, it is the responsibility of public health experts to educate patients regarding maternal care through counseling programs and seminars to enhance the mother’s knowledge regarding maternal care.

## 5. Implications for Practice and/or Policy

This study has practice implications for both practitioners and policymakers. Firstly, the sub-groups of gestational age and other CS-associated risk factors should be addressed by the practitioners and policymakers to reduce the CS frequency and adhere to the WHO recommendations. Secondly, education can change patients’ decisions regarding maternal care and CS. Therefore, targeted educational interventions should be designed and implemented at community levels to improve maternal care through the eradication of myths and misconceptions about the NVD.

## 6. Limitation

The initial sample size for this study using the Daniel formula was 288 [58], but due to COVID-19 pandemic and the sudden national lockdown, the hospital administration denied access to patients for data collection. Therefore, the study was terminated and data of only 248 patients were collected. Therefore, the sample size in the current study was small, and the results cannot be generalized to the whole population. Secondly, the questionnaire was mainly focused on assessing the frequency of CS and its associated risk factors, and it may not have covered all the aspects associated with CS.

## 7. Conclusions

The current study explained the risk factors that are associated with increasing CSR. The highlighted risk factors were preterm of gestational age, mothers of a younger age (20–24 years), and mothers that belong to urban populations. These risk factors can be addressed by focused educational interventions and regular hospital visits during their gestational period. Patient and caregiver education regarding maternal care can change the patient’s decision regarding CS, and attention should be paid to educate medical personnel for recommending the CS as per given guidelines. Moreover, the already implemented national family planning and chief care programs should be reshaped and extended to urban and rural areas to improve maternal care. The health regulatory agencies in Pakistan should implement evidence-based practice guidelines along with compliance to the recommended CS indications for addressing the overwhelming increase in CS frequency.

## Figures and Tables

**Table 1:** The association of demographic variables with the mode of delivery.

Demographic Variables	Total = 248		Cesarean Birth = 173		Normal Birth = 75		*p*-Value
	N	%	N	%	N	%	
Age (yrs.)							0.03
20–24	79	31.8	64	81.0	15	18.9	
25–29	71	28.6	49	69.0	22	30.9	
30–34	71	28.6	42	59.2	29	40.8	
≥35	27	10.8	20	74.1	07	25.9	
Gestational Age (weeks)							<0.001
Pre-term (<37)	135	54.4	109	80.7	26	19.26	
Late-term (34–36)	16	6.5	15	93.7	1	0.6	
Early-term (37–38)	56	22.6	32	57.1	24	42.8	
Full-term (39–40)	39	15.7	17	43.5	22	56.4	
Post-term (≥40)	02	0.08	02	100	0	0	
Area of Residence							0.05
Urban	169	68.1	126	74.5	43	25.4	
Rural	79	31.8	49	62.0	30	37.9	
Education level (grades)							0.001
Un-educated	48	19.3	24	50.0	24	50.0	
Primary (1–6)	44	17.7	32	72.7	12	27.3	
Secondary (7–12)	56	22.6	45	80.3	11	19.6	
Tertiary (Bachelors)	70	28.2	57	81.4	13	18.6	
Higher (Masters)	30	12.1	17	56.6	13	43.3	
Living pattern							0.001
Living alone	153	61.7	120	78.4	33	21.5	
With family	95	38.3	55	57.9	40	42.1	
Physical Activity							<0.001
High	53	21.4	23	43.4	30	56.6	
Moderate	107	43.1	73	68.2	34	31.7	
Low	88	35.5	79	89.7	09	10.2	
Parity							0.01
Low multi-parity (1–3)	182	73.4	121	66.5	61	33.5	
Grand multi-parity (4–8)	66	26.6	54	81.8	12	18.2	
Gravidity							0.12
Primi-gravida (1)	100	40.3	66	66.0	34	34.0	
Multi-gravida (>1)	148	59.7	109	73.6	39	26.3	
Hookah user							0.76
Current user	4	1.6	3	75	1	25	
Former user	11	4.4	8	72.7	3	27.2	
Never used	233	93.9	164	70.4	69	29.6	

Fisher exact test was applied when the cell counts were less than 5.

**Table 2:** The association of pregnancy complications with the mode of delivery.

Obstetric Variables	Total = 248		Cesarean Birth = 173		Vaginal Birth = 75		*p*-Value
	N	%	N	%	N	%	
Pregnancy complications							<0.001
None	168	67.7	97	57.7	71	42.2	
Gestational diabetes	06	2.4	06	100	00	00	
Hypertension	10	4.0	10	100	00	00	
Pre-eclampsia/eclampsia	13	5.2	13	100	00	00	
hypothyroidism	36	14.5	34	94.4	02	5.5	
Significant vaginal bleeding	13	5.2	13	100	00	00	
Others	02	0.8	00	00	02	100	

Fisher exact test was applied when the cell counts were less than 5.

**Table 3:** Classification of CS based on Royal College of Obstetricians and Gynecologists (RCOG) categorization.

RCOG Categories	Frequency (n)	Percentage (%)
Category 1	38	19.8%
Category 2	5	2.6%
Category 3	70	36.5%
Category 4	79	41.1%

**Table 4:** Knowledge regarding CS in the study population.

Question		Frequency	Percentage
Have you ever heard about CS?	Yes	247	99.5
	No	1	0.40
	Do not know	0	0
Do you think you have enough knowledge about CS?	Yes	156	62.9
	No	92	37.1
	Do not know	0	0
Do you know vaginal delivery after CS was feasible?	Yes	72	29.0
	No	141	56.9
	Do not know	35	14.1
Do you know that blood could be needed during or after the operation?	Yes	124	50
	No	124	50
	Do not know	0	0
Do you think that CS is hazardous?	Yes	170	68.5
	No	60	24.2
	Do not know	18	7.26
Do you agree CS will cause death?	Yes	68	27.4
	No	138	55.6
	Do not know	42	16.9
Do you agree vaginal birth can cause death?	Yes	74	29.8
	No	137	55.2
	Do not know	37	14.9
Do you prefer CS on the doctor’s advice?	Yes	248	100
	No	0	00
	Do not know	0	00
Do you agree that vaginal birth can cause pain?	Yes	248	100
	No	0	00
	Do not know	0	00
Do you agree CS must be discouraged because of post-surgical pain?	Yes	171	68.9
	No	20	8.0
	Do not know	56	22.5
Do you believe CS is the safest way to save the lives of mothers and babies?	Yes	189	76.2
	No	03	1.2
	Do not know	56	22.5
Would you believe a cesarean could lead to infertility?	Yes	61	24.5
	No	11	4.4
	Do not know	156	62.9
Do you agree that CS is related to previous CS?	Yes	216	87.0
	No	14	5.6
	Do not know	18	7.2
Do you believe CS is secure than normal birth?	Yes	24	9.6
	No	201	81.0
	Do not know	23	9.2

## Data Availability

The data can be requested from the corresponding author on a valid reason.

## Supplementary Materials

The following are available online at https://www.mdpi.com/article/10.3390/ijerph18168812/s1, Table S1: The study tool.
