# Supplementary material for: A Cross-Sectional Study to Assess the Frequency and Risk Factors Associated with Cesarean Section in Southern Punjab, Pakistan
Source: Int J Environ Res Public Health. 2021 Aug 21;18(16):8812. doi: 10.3390/ijerph18168812 (PMC8391541; doi:10.3390/ijerph18168812)
Supplement: Supplementary file 1 [file ijerph-18-08812-s001.zip › ijerph-1282947-supplementary.pdf]

Supplementary information

A cross-sectional study to assess the frequency and risk factors associated with cesarean section in Southern Punjab, Pakistan

Muhammad Fawad Rasool[1*], Saira Akhtar[1], Iltaf Hussain[1], Abdul Majeed[1], Imran Imran[2], Hamid Saeed[3], Muqarrab Akbar[4], Muhammad Omer Chaudhry[5], Anees Ur Rehman[1], Waseem Ashraf[2], Faleh Alqahtani[6*], Hussain Alqhtani[7]

Supplementary Table S1. The study tool

**Socio-demographic factors**	**Responses**			
**Age group (years)**	16-25	26-35	>36	
**Gestational age (weeks)**	Preterm (37 or below)	Full-term (39–40)	Late-term (41)	Post-term (40 or above)
**Education**	Primary	Secondary	Tertiary	
**Place of Residency**	Urban	Rural		
**Societal life**	Living alone	Living with family		
**Physical Activity**	High activity	Moderate activity	Low activity	
**Hookah user**	Yes	No		
**Parity (number of births given after 20 weeks of gestation)**	Nulli-parity (0)	Low multi-parity (1-3)	Grand multi-parity (4-8)	
**Gravidity (total number of confirmed pregnancies, regardless of outcome)**	Nulli-gravida (0)	Primi-gravida (1)	Multi-gravida (>1)	

**Obstetric variables**					
Pregnancy complications	Hypertension	Pre-eclampsia/ eclampsia	Thyroid problem	Significant vaginal bleeding	Other

**Indication for cesarean section**							
Dystocia (obstructed labor, long labor, failed induction of labor)	Breech presentation	Transverse presentation	Fetal distress (abnormal fetal heart rate, cord prolapsed)	Placenta	praevia/ accreta	other	Past cesarean history

Knowledge about cesarean section			
**Question items**	**Responses**		
Have you ever heard about CS?	Yes	No	Don’t know
Do you think you have enough knowledge about CS?	Yes	No	Don’t know
Do you know vaginal delivery after CS was feasible?	Yes	No	Don’t know
Do you know that blood could be needed during or after the operation?	Yes	No	Don’t know
Do you consider that CS is dangerous?	Yes	No	Don’t know
Do you agree CS will cause death??	Yes	No	Don’t know
Do you agree vaginal birth will cause death?	Yes	No	Don’t know
Do you prefer CS on doctor's advice?	Yes	No	Don’t know
Do you agree that vaginal birth can cause pain?	Yes	No	Don’t know
Do you agree CS must be discouraged because of post-surgical pain?	Yes	No	Don’t know
Do you believe CS is the safest way to save the lives of mothers and babies?	Yes	No	Don’t know
Would you believe a cesarean could lead to infertility?	Yes	No	Don’t know
Do you agree that CS is related to previous CS?	Yes	No	Don’t know
